# Supplementary material for: Repurposing Anti-diabetic Drugs to Cripple Quorum Sensing in Pseudomonas aeruginosa
Source: Microorganisms. 2020 Aug 22;8(9):1285. doi: 10.3390/microorganisms8091285 (PMC7569791; doi:10.3390/microorganisms8091285)
Supplement: Supplementary file 1 [file microorganisms-08-01285-s001.pdf]

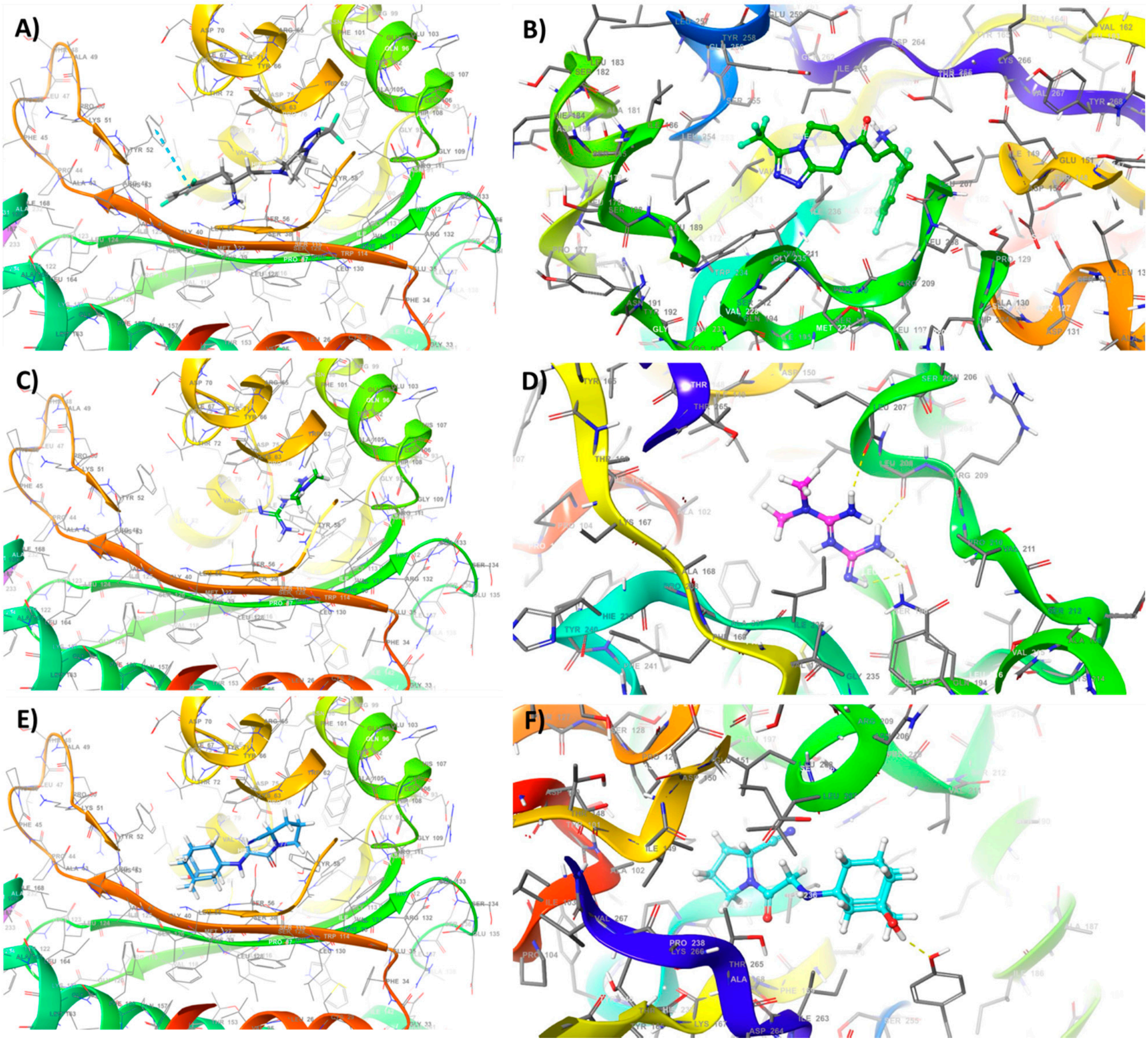

Supplementary Figure S1. Docking study of tested anti-diabetic drugs into *P. aeruginosa* QS receptors QscR and PqsR. QscR receptor 3D binding mode of (**A**) sitagliptin (gray), (**C**) metformin (green) and (**E**) vildagliptin (blue). 3D binding mode of (**B**) sitagliptin (green) with (**D**) metformin (burble) and (**F**) vildagliptin (greenish) with amino acids in the active sites of pqsR. .
